# Mindfulness and impulsive behavior: exploring the mediating roles of self-reflection and coping effectiveness among high-level athletes in Central China

**DOI:** 10.3389/fpsyg.2024.1304901

**Published:** 2024-01-11

**Authors:** Peili Liang, Hongyu Jiang, Huilin Wang, Jiaxin Tang

**Affiliations:** ^1^School of Physical Education, Hunan University of Science and Technology, Xiangtan, China; ^2^School of Business, Hunan University of Science and Technology, Xiangtan, China; ^3^Moray House School of Education, The University of Edinburgh, Edinburgh, United Kingdom

**Keywords:** athlete, mindfulness, self-reflection, coping effectiveness, impulsive behavior

## Abstract

**Introduction:**

In the highly competitive field of sports, impulsive behavior by athletes not only threatens personal and team harmony but also poses significant risks to their careers and public image. Despite these behaviors often becoming the focus of public attention, their underlying causes and prevention strategies remain relatively unknown. This study delves deep into the impact of mindfulness on athletes’ impulsive behavior, revealing the mediating roles of self-reflection and coping effectiveness.

**Methods:**

Using a combination of snowball and convenience sampling, a sample of 403 athletes from high-level sports teams in the Central China region participated in a questionnaire survey. The data were analyzed using Amos v.23 software.

**Results:**

The findings indicate a positive correlation between mindfulness and coping effectiveness (standardized coefficient = 0.336, *p* < 0.001), as well as between self-reflection and coping effectiveness (standardized coefficient = 0.406, *p* < 0.001). There is a negative correlation between coping effectiveness and impulsive behavior (standardized coefficient = −0.476, *p* < 0.001). The positive impact of mindfulness on impulsive behavior (standardized coefficient = −0.371, *p* < 0.01) is mediated by self-reflection and coping effectiveness. The explanatory power of this study is R^2^ = 0.35.

**Discussion:**

Mindfulness reduces impulsive behavior by enhancing self-reflection capabilities and improving coping effectiveness. Based on these substantive research results, to mitigate impulsive behavior in athletes, it is recommended that the National Sports Administration and coaches actively implement mindfulness training. Additionally, targeted psychological intervention strategies should be developed to enhance athletes’ mental health levels and optimize their sports performance.

## 1 Introduction

In the field of sports, athletes continuously push the boundaries of their physical capabilities and mental resilience. The intricate interplay between behavior and performance has always been a subject of keen exploration. Athletes, embodying discipline and dedication (Spiotta et al., 2018), relentlessly strive to enhance their athletic achievements and attain excellence, making it one of their foremost objectives (Dehghani et al., 2018). The lives of athletes are closely intertwined with the competitive arena, where they undergo rigorous training, endure physical ailments, and make sacrifices in their family and social lives, all in the pursuit of sporting perfection and outstanding achievements. However, this pursuit of excellence, at times, poses emotional and behavioral challenges, especially when athletes face the pressures and uncertainties of competitive sports.

Amidst the spotlight of victory and honor, a subtler aspect of their behavior has drawn the attention of researchers and practitioners – impulsive behavior. It is well-known that competitive sports often come with intense rivalry and immense pressure (Silva, 1990). This competitive nature in the sports domain ignites athletes’ intrinsic ambitions and determination, driving them to continually surpass their limits for outstanding performance. However, accompanying this fierce competition are concerning issues, such as how athletes should cope with stress (Diotaiuti et al., 2021) and shield themselves from the impact of stressors (Diotaiuti et al., 2021), particularly regarding athletes’ impulsive behavior, a problem that has garnered widespread attention among researchers (Parent and Fortier, 2017). Athletes’ impulsive behaviors, especially those characterized by violence and aggression, both on and off the field, have been regarded as a societal issue (Tenenbaum et al., 1997). Moreover, most research on athlete violence and aggression has focused on the negative aspects of these behaviors (Kimble et al., 2010). This not only affects the sports domain but also has adverse effects on societal values and culture.

Relevant studies suggest that athletes of the same age group are more prone to aggression than non-athletes (Lenzi et al., 1997), indicating that athletes are more susceptible to impulsive behaviors. Such impulsive behaviors can tarnish the image of athletes in the public eye, leading to negative impressions among the public and the media (Brown et al., 2018). Athlete impulsive behavior extends beyond the realm of competition (Tenenbaum et al., 1997), challenging not only the behavioral codes upheld by sports organizations but also the essence of fair competition and mutual respect that underpins the spirit of sportsmanship. Therefore, it is necessary to explore methods to mitigate athlete impulsive behavior, with a foundation built on sound mental health.

Research has shown that mindfulness can enhance emotional regulation (Teper et al., 2013) and benefit mental health. In the realm of sports, mindfulness has been widely applied to improve athletes’ psychological well-being (Anderson et al., 2021). Some studies have indicated that athletes’ impulsive tendencies can be improved through interventions like mindfulness meditation (Sánchez-Sánchez et al., 2023). Although sport impulsion differs from athlete impulsive behavior, the research findings offer valuable insights for this study.

Previous research on athlete impulsive behavior has primarily focused on factors such as athlete’s competitive anxiety (Terres-Barcala et al., 2022), psychological traits (Gabrys and Wontorczyk, 2022), technical performance (Lage et al., 2011), and also included studies on athletes with sports-related concussions (Liebel et al., 2021; Byrd et al., 2022). The academic community has not thoroughly explored the relationship between athlete mindfulness and impulsive behavior. Additionally, shifting the research focus to Chinese athletes is expected to provide more specific and applicable results for the prevalent issues in the Chinese sports environment. Unlike previous research, this study delves into the potential relationship between athlete mindfulness and impulsive behavior, with a particular emphasis on the mediating roles of coping effectiveness and self-reflection. The former aids athletes in effectively dealing with various competitive pressures, while the latter assists athletes in gaining a deeper understanding of their thoughts, emotions, and behaviors. The interplay of these factors is crucial for understanding the formation and regulation of athlete impulsive behavior.

Given the gaps and issues in the aforementioned research landscape, this study aims to: (1) explore the relationships between mindfulness, self-reflection, coping effectiveness, and impulsive behavior to uncover potential mutual influences and interactions; (2) investigate potential synergies between mindfulness, self-reflection, and coping effectiveness, emphasizing their importance in athlete mental health; (3) ultimately provide targeted recommendations to reduce athlete impulsive behavior, promoting the healthy development of the sports domain.

This study centers on athlete impulsive behavior and proposes mindfulness as an intervention to improve it. The results suggest that self-reflection and coping effectiveness mediate the relationship between mindfulness and impulsive behavior. Mindfulness interventions enhance athletes’ self-reflection and coping effectiveness, leading to a reduction in impulsive behavior. Therefore, understanding the impact of mindfulness on athlete impulsive behavior not only contributes to the theoretical development in relevant fields and societal concerns but also promotes athletes’ outstanding performance in competitive sports, safeguarding their mental health and reducing the negative impact of impulsive behavior on both individuals and society as a whole. Additionally, it helps society better understand and support the psychological needs of athletes, playing a crucial role in shaping a positive sports culture and raising the ethical standards of the sports industry.

## 2 Literature review and hypothesis development

### 2.1 The concept of variables

#### 2.1.1 Mindfulness

The term mindfulness originates from the Pali word “sati” in ancient texts, which translates to awareness, attention, and memory (Siegel et al., 2009). Over time, the definition of mindfulness has been appropriately adapted in the fields of psychology and medicine, encompassing a broader range of meanings. Baer (2003) proposed that mindfulness is linked to the unique attributes of attention and consciousness and can be cultivated through meditation. Building upon Kabat-Zinn (2003), a pioneer in the application of mindfulness-based therapies, defined mindfulness as the awareness that arises through paying attention, on purpose, in the present moment, non-judgmentally, to the unfolding of experience. Bishop et al. (2004) expanded on this definition, stating that mindfulness starts with regulating the focus of attention, bringing consciousness to present-moment experiences, and observing and attending to the continually changing thoughts, emotions, and sensations.

Given that this study focuses on athletes, the definition of mindfulness employed in this research encompasses a mental state and conscious attitude. It emphasizes being fully present in the moment, attentively experiencing and being aware of one’s sensations, thoughts, emotions, and the surrounding environment, all without judgment or bias.

#### 2.1.2 Self-reflection

Self-reflection is regarded as a fundamental human trait and a cornerstone of advanced psychological functioning. Hilzensauer (2008), approaching it from a perspective of reflective learning, posits that self-reflection is the ability to connect personal strengths and limitations, take action with a critical attitude, and become aware of the challenges or potential opportunities in one’s learning process. Lew and Schmidt (2011), looking at it through the lens of students and academic performance, describe self-reflection as a process where a learner reviews their past learning experiences, the actions they took to facilitate learning, and explores the connection between the knowledge taught and their own perceptions of that knowledge.

Drawing on the definition provided by Grant, Franklin (Grant et al., 2002), this study focuses on self-reflection in the context of athletes. Athletes are independent individuals, and therefore, in this research, we adopt Grant et al.’s definition of self-reflection, which involves examining and evaluating one’s thoughts, emotions, and behaviors.

#### 2.1.3 Coping effectiveness

Exceptional athletes require a set of coping skills to effectively deal with sources of pressure (Dugdale et al., 2002). In various athletic performances, an athlete’s inability to cope effectively with stressors is considered one of the significant factors that can hinder their optimal performance (Lazarus, 2000). The concept of coping effectiveness has not received widespread research attention (Ntoumanis and Biddle, 1998). Nicholls, Holt (Nicholls et al., 2005) suggest that coping effectiveness can be evaluated based on short-term outcomes, such as an individual’s ability to cope with specific stressors, or long-term outcomes, such as their overall adaptation over time. Folkman and Moskowitz (2004) argue that coping effectiveness needs to be assessed in relation to its application in specific stress situations, as a particular strategy may be effective in one context but ineffective in another. In summary, within the context of this study, coping effectiveness is defined as an individual’s ability to cope with stressors, challenges, or adversities in a way that promotes emotional stability, psychological balance, and positive adaptation.

#### 2.1.4 Impulsive behavior

Moeller, Barratt (Moeller et al., 2001) defined impulsivity as a tendency to react rapidly and without planning to internal or external stimuli, regardless of the negative consequences of these responses. Impulsive behaviors encompass a wide range of actions, including but not limited to alcohol dependence (Dick et al., 2010), substance abuse (Perry and Carroll, 2008; Argyriou et al., 2018), and antisocial behavior (Luengo et al., 1994). As this study focuses on athletes, the definition of impulsive behavior employed in this research draws from Moeller et al.’s definition, which characterizes impulsive behavior as quick, unplanned reactions to internal or external stimuli, without consideration for the negative consequences of these responses.

### 2.2 Hypotheses

#### 2.2.1 Mindfulness, self-reflection, and coping effectiveness

Mindfulness, in its traditional understanding, refers to purposefully focusing on the present moment without judgment (Lewis et al., 2022). Previous research has shown that mindfulness can cultivate self-awareness (Friese et al., 2012; Lawlor, 2016; Berkovich-Ohana et al., 2019). This cultivated awareness deepens the observation and attention to one’s thoughts, emotions, and bodily sensations. When athletes possess heightened mental awareness, they can observe their thoughts and emotions without becoming entangled in them, creating a conducive environment for self-reflection (Mohebi et al., 2022). Self-reflection involves the capacity for introspection and objective analysis of one’s thoughts, behaviors, and decisions. In a sense, mindfulness forms the foundation for self-reflection by providing the necessary clarity and distance from immediate reactions (Wang et al., 2023).

In sports, athletes often need to analyze their performances, understand their mistakes, and adjust their strategies. Thus, by promoting mindfulness, athletes are more likely to engage in self-reflective behavior. Research suggests that Mindful-ness-Acceptance-Commitment (MAC) interventions emphasize acceptance and non-reactivity, enabling individuals to cope with stress without being overwhelmed (Wong et al., 2022). Athletes frequently encounter high-pressure situations and substantial physical and mental challenges, from which they can benefit by cultivating this calmness. Coping effectiveness refers to the ability to manage and deal with stress in a way that promotes well-being and functional outcomes (Kaiseler et al., 2017). Mindfulness, by promoting non-reactivity, reduces impulsivity and potential harmful reactions, enhancing an individual’s ability to cope with stress (Zou et al., 2020). Moreover, in a mindful state, athletes can choose how to respond to challenges rather than automatically react, making their coping strategies more deliberate and effective.

Self-reflection is the process of introspection and analysis of one’s actions, decisions, and experiences (Kaiseler et al., 2012). Athletes who engage in regular self-reflection can better understand their sources of stress, identify triggers, and implement appropriate coping strategies (Dagnall et al., 2021). Through self-reflection, athletes gain insight into their strengths and weaknesses, making it easier to adopt the most effective coping strategies in any situation. Additionally, self-reflection enhances self-awareness, enabling athletes to proactively manage potential stressors or handle stress with higher skill (Modena et al., 2022). Over time, this proactive and thoughtful approach to facing challenges can improve an athlete’s overall coping effectiveness. Hence, this study proposes the following hypotheses:

*H1*: Mindfulness has a positive and significant influence on self-reflection.

*H2*: Mindfulness has a positive and significant influence on coping effectiveness.

*H3*: Self-reflection has a positive and significant influence on coping effectiveness.

#### 2.2.2 Self-reflection, coping effectiveness and impulsive behavior

Impulsivity is typically characterized by a lack of careful consideration and an inability to resist immediate temptations (Gabrys and Wontorczyk, 2022). On the other hand, self-reflection promotes a deeper understanding of one’s actions, decisions, and motivations (González-Hernández et al., 2019). When athletes engage in self-reflection, they often gain a clear awareness of their past behaviors and the consequences of those actions. By continuously evaluating their choices and understanding the outcomes of impulsive behavior, they can better resist impulsive actions in the future. Additionally, self-reflection enhances awareness of one’s own emotions and triggers (Kemp et al., 2019). Athletes who engage in self-reflection can identify situations or emotions that might lead to impulsive reactions, allowing them to proactively address these triggers or approach them with greater caution. The act of self-reflection itself requires slowing down and thinking, serving as a counterbalance to impulsive tendencies.

The underlying mechanisms that trigger impulsive behavior may stem from various neurocognitive and environmental factors, but their manifestation can be significantly modulated through coping mechanisms. In the field of sports psychology, the role of coping effectiveness is crucial, influencing an athlete’s performance trajectory and overall well-being (Smith, 1986). The higher one’s coping effectiveness, the better equipped they are to make informed assessments and responses to challenging situations, minimizing the tendency for impulsive reactions. Faced with an array of stimuli, whether they arise from opponent provocations or dynamic changes during a game, athletes with honed coping mechanisms can engage in cognitive assessments, ensuring that their reactions align with the immediacy of the situation and the overarching objectives (Gross, 1998). This cognitive mediation serves as a counter to impulsive tendencies, involving the evaluation of event significance, potential consequences of various responses, and the selection of the most appropriate course of action. This cognitive mediation can mitigate the immediacy of impulsive responses, promoting more thoughtful reactions that align with broader personal goals and situational contexts.

Furthermore, research suggests that effective coping strategies, such as problem-focused coping, emotion-focused coping, and utilizing social support, are associated with reducing impulsive tendencies under stressful conditions (Compas et al., 2001). After cultivation and implementation, these strategies enhance athletes’ cognitive abilities, enabling them to navigate situations that might trigger impulsive behavior. Therefore, this study proposes Hypothesis 4 and Hypothesis 5:

*H4:* Self-reflection has a negative and significant impact on impulsive behavior.

*H5:* Coping effectiveness has a negative and significant impact on impulsive behavior.

#### 2.2.3 The mediating effects

Mindfulness is rooted in present-moment awareness, providing athletes with a cognitive foundation to observe thoughts and emotions without immediately reacting. Research indicates that mindfulness practices can enhance attention control, emotional regulation, and self-awareness, laying the groundwork for subsequent behavioral processes (Bishop et al., 2004). Building upon the heightened awareness facilitated by mindfulness, self-reflection offers a conscious analysis of one’s internal state. Moreover, engaging in self-reflection aids in understanding personal motivations, actions, and the potential consequences of those actions (Ryan and Deci, 2006). In the realm of sports, the combination of coping strategies with the ability to choose optimal responses and take corresponding actions can thwart impulsive behavior (Smith, 1986). Effective coping strategies can mitigate the impact of stressors that may trigger impulsive behavior. For instance, athletes with effective coping strategies may avoid impulsive decisions in high-pressure situations to prevent adverse effects on their performance (Nicholls and Polman, 2007). Therefore, this study proposes the following mediation hypothesis:

*H6*: Self-reflection and coping effectiveness mediate the relationship between mindfulness and impulsive behavior.

**Figure 1:**
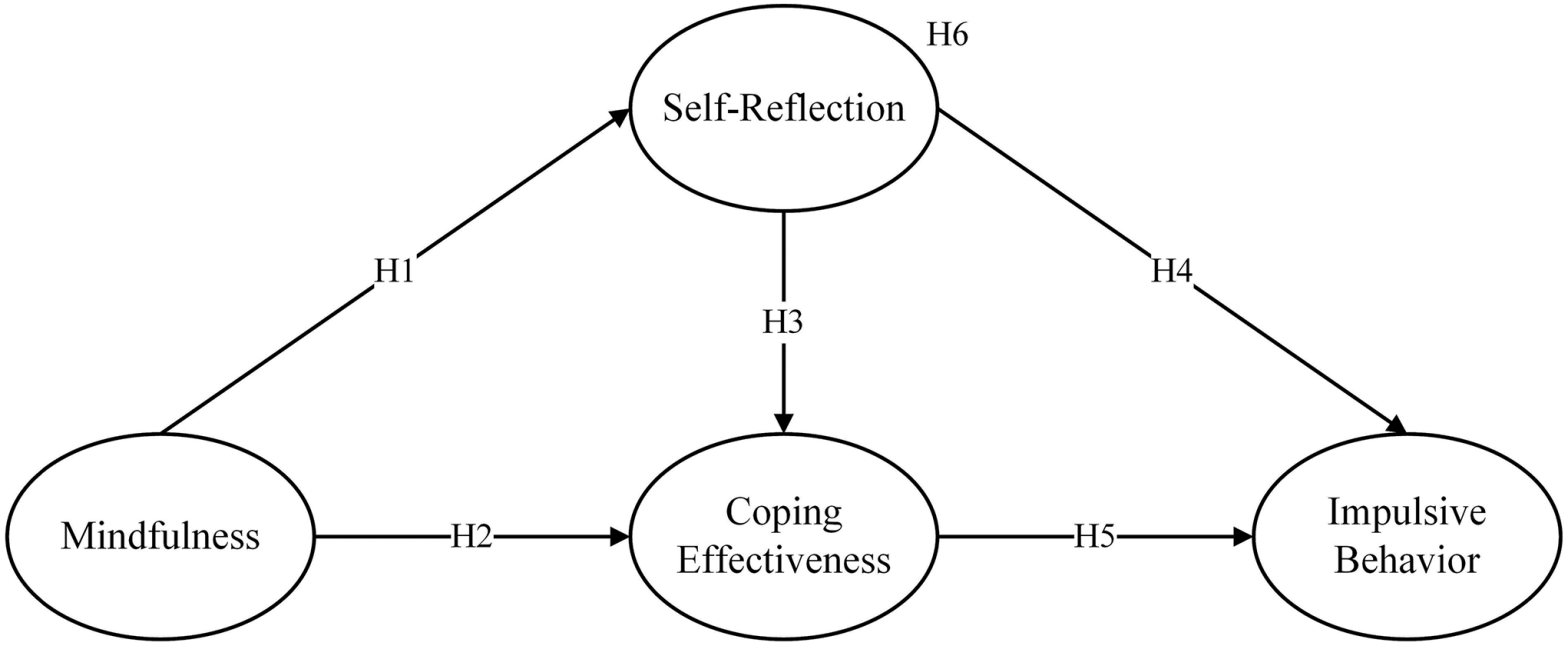
The hypothesized model.

All hypotheses are depicted in Figure 1.

## 3 Materials and methods

### 3.1 Participants and procedure

This study employed a combination of snowball sampling and convenience sampling methods, targeting athletes from the Central China region. Researchers conducted a questionnaire survey on sports athletes in the Central China region from June to July 2023. To ensure the reliability of the data, researchers established contact with high-level sports team leaders from different provinces in the Central China region. After obtaining approval from these team leaders, the survey questionnaires were distributed to the athletes. Before completing the questionnaires, all respondents received explanations about the purpose of participating in the survey, signed informed consent forms, and pledged to keep the survey results strictly confidential. Based on this, the snowball method was used, and eligible team members shared the survey questionnaires among themselves. Upon completing the questionnaires, the researchers provided some small gifts (e.g., sports gloves, wristbands, knee pads, and other sports equipment) to express gratitude to the respondents. A total of 600 questionnaires were distributed, and by the end of July, 482 athletes had been surveyed. After excluding invalid questionnaires (due to abnormal data, missing information, blank responses, etc.), a total of 403 valid questionnaires were obtained, resulting in an effective response rate of 67.2%.

Table 1 presents the demographic characteristics of the 403 surveyed athletes. Among the respondents, (1) 60.3% were between the ages of 18 and 23; (2) males accounted for 64.5%, while females accounted for 35.5%; (3) team sports were the primary sports discipline (48.4%); (4) the majority of respondents (68.2%) had participated in sports competitions 1–3 times in the past 12 months. This data might be skewed due to the official lifting of COVID-19 prevention and control measures in China on January 8, 2023, which meant that sports competitions could not take place normally before that date, and athletes were unable to participate in such events.

**Table 1:** Participant profile (*N* = 403).

Profiles		Survey (%)
Respondents’ age	<18	8.7
	18–20	24.1
	21–23	36.2
	>23	31.0
Respondents’ gender	Male	64.5
	Female	35.5
Respondents’ sports items	Ball sports	48.4
	Athletics projects	17.4
	Other sports	34.2
Respondents’ sports events in the last 12 months	1–3	68.2
	4–6	14.7
	7–9	5.7
	>10	11.4

### 3.2 Measures

The questionnaire consisted of five sections. The first section required respondents to report demographic information, including age, gender, the sports discipline they were involved in, and the number of sports competitions they had participated in over the past 12 months. The second section collected data on mindfulness from the respondents, using five items from the scale developed by Feldman, Hayes (Feldman et al., 2007). Sample items included “It is easy for me to concentrate on what I am doing.” The third section gathered data on respondents’ coping effectiveness, measured using four items from the scale developed by Gottlieb and Rooney (2004). Sample items included “I’m dealing with this problem better now than I used to.” The fourth section collected data on respondents’ self-reflection, measured using four items from the scale developed by Naeimi, Abbaszadeh (Naeimi et al., 2019). Sample items included “I often think about the way I feel about things.” Finally, the fifth section gathered data on respondents’ impulsive behavior, measured using four items from the scale developed by Whiteside and Lynam (2001). Sample items included “Sometimes I do things on impulse that I later regret.” All of the above scales were measured using a Likert five-point scale, with response options ranging from 1 (strongly disagree) to 5 (strongly agree).

To adapt the scales to the Chinese cultural context and language, some items in the scales were modified by the researchers. Therefore, a pilot test was conducted to ensure the reliability of the modified scales (Kimberlin and Winterstein, 2008). The pilot test received a total of 96 valid questionnaires, and the results showed that Cronbach’s alpha coefficients were all greater than 0.8, indicating that the researchers’ appropriate modifications to the scales were reasonable and reliable.

### 3.3 Data analysis

This study utilized AMOS v.23 to construct a Structural Equation Model (SEM) aimed at examining the enhancement of impulse behavior through mindfulness among athletes. Model parameter estimation was conducted using the Maximum Likelihood (ML) estimation method. A two-step modeling approach was employed to assess both the measurement model and the structural model. Initially, a comprehensive evaluation of the model’s reliability and validity was performed. Subsequently, fit indices and path coefficients of the hypothesized model were measured, along with an examination of the presence of mediating effects.

To mitigate potential issues stemming from common method variance (CMV) inherent in self-reported behaviors, the approach recommended by Mossholder, Bennett (Mossholder et al., 1998) was adopted by the researchers. In line with this, a comparison was undertaken between model one and model two, with a focus on assessing variations in degrees of freedom and chi-square values. The outcomes revealed that the chi-square value for model one amounted to 3345.003, encompassing 119 degrees of freedom and yielding a value of *p* below 0.001. Correspondingly, the chi-square value for model two stood at 230.992, accompanied by 113 degrees of freedom and a value of p below 0.001. These findings affirm the proportional fit of model one to model two. Hence, it can be deduced that the absence of evidence supporting a univariate structure suggests the absence of concerns related to CMV within the scope of this study.

## 4 Results

### 4.1 Measurement model

The assessment of latent variables’ reliability and validity involved the implementation of confirmatory factor analysis (CFA) utilizing AMOS v.23. All variables exhibited Cronbach’s α values above 0.9 (refer to Table 2), affirming the robust internal consistency within the model structure (Fornell and Larcker, 1981). Furthermore, the average variance extraction (AVE) for each variable surpassed 0.6 (as indicated in Table 2), and the composite reliability (CR) of every latent variable exceeded 0.8, thereby attesting to the model’s strong convergent validity. The robustness of convergence validity across the proposed models was well established. Factor loadings derived from principal component factor analysis ranged from 0.725 to 0.942 (see Table 2), underscoring the robust construct validity of the measurement model. Discriminant validity was established, as evidenced by the square root of the AVE along the diagonal exceeding the correlation between constructs (refer to Table 3).

**Table 2:** Reliability and validity.

Items	Loadings	Cronbach’s α	CR	AVE
*Mindfulness (MI)*		0.943	0.944	0.770
MI1	0.884			
MI2	0.839			
MI3	0.900			
MI4	0.877			
MI5	0.886			
*Self-reflection (SR)*		0.958	0.958	0.852
SR1	0.909			
SR2	0.942			
SR3	0.925			
SR4	0.915			
*Coping effectiveness (CE)*		0.905	0.898	0.691
CE1	0.725			
CE2	0.732			
CE3	0.923			
CE4	0.922			
*Impulsive behavior (IB)*		0.937	0.937	0.788
IB1	0.902			
IB2	0.862			
IB3	0.913			
IB4	0.873			

**Table 3:** Pearson correlation.

Construct	MI	SR	CE	IB
MI	(0.877)			
SR	0.559 **	(0.923)		
CE	0.510 **	0.529 **	(0.831)	
IB	−0.416 **	−0.430 **	−0.523 **	(0.888)

The square root of the AVE is in diagonals; off diagonals are a person’s corrections of contracts. **p < 0.01.

### 4.2 Structural model

**Figure 2:**
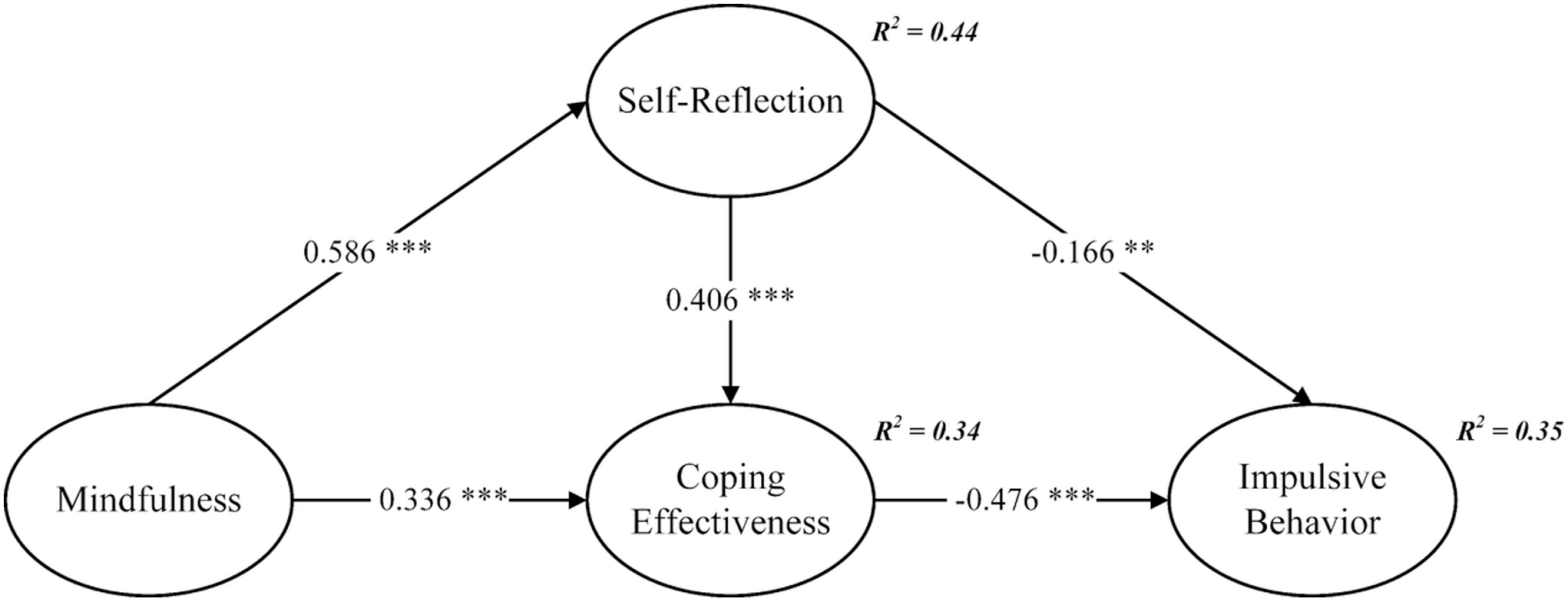
Structural path model. **p < 0.01, ***p < 0.001.

Upon scrutinizing the measurement model’s reliability and validity, this investigation proceeded to analyze the structural model through the utilization of AMOS v.23 in order to substantiate the hypotheses. The outcomes of CFA, employing 5,000 bootstrap samples, consistently align with the recommended criteria (*χ*^2^/df = 2.739, GFI = 0.918, NFI = 0.955, TLI = 0.965, CFI = 0.971, RMSEA = 0.066), thus signifying an optimal fit between the model and the empirical data. Moreover, the outcomes of Pearson correlation are outlined in Table 3, corroborating the interconnectedness of the variables. The standardized coefficients for the structural equation model’s variables are depicted in Figure 2. As illustrated in Figure 2, mindfulness exhibited a direct and positive correlation with self-reflection (*β* = 0.586, *p* < 0.001) and coping effectiveness (*β* = 0.336, *p* < 0.001), substantiating H1 and H2, respectively; self-reflection demonstrated a direct and affirmative correlation with coping effectiveness (*β* = 0.406, *p* < 0.001), thus supporting H3; concurrently, self-reflection exhibited a direct and adverse association with impulsive behavior (*β* = −0.166, *p* < 0.01), affirming H4; lastly, coping effectiveness was found to directly and negatively correlate with impulsive behavior (*β* = −0.476, *p* < 0.001), substantiating H5.

As Table 4 illustrates, the examination of mediating effects was carried out using bootstrap estimation with 5,000 resamples and 95% bias-corrected confidence intervals. The results distinctly underscore that the indirect effect of mindfulness on impulsive behavior, mediated through self-reflection and coping effectiveness, amounted to −0.371 [SE = 0.042, CI = (−0.450, −0.285), *p* < 0.01], thus offering robust support for H6.

**Table 4:** Standardized indirect effect.

	Point estimate	Product of coefficients		Bootstrapping		
				Bias-corrected 95% CI		Two-tailed significance
		SE	Z	Lower	Upper	
MI → IB	−0.371	0.042	−8.833	−0.450	−0.285	0.01 (**)

## 5 Discussion

### 5.1 Theoretical contribution

This study contributes significantly to the theoretical analysis of impulsive behavior in athletes, making several noteworthy contributions. Firstly, prior research on athlete impulsivity has predominantly focused on aspects such as competitive anxiety (Terres-Barcala et al., 2022) and psychological characteristics (Gabrys and Wontorczyk, 2022). However, there has been limited exploration into the specific nature of athlete impulsive behavior. This study takes a novel approach by concentrating on the determinants of athlete impulsive behavior, offering a more targeted and enriched perspective within the existing theoretical framework. The researchers believe that by investigating athlete impulsive behavior, they can identify critical factors that influence it, and subsequently propose solutions and interventions. Like previous studies, it is acknowledged that athletes are prone to impulsive behavior (Lenzi et al., 1997). However, this research goes a step further in asserting that the occurrence of athlete impulsive behavior is closely related to how athletes handle stress, especially considering the high-pressure environments inherent in their professional roles, which can impact their psychological resilience (Iso-Ahola, 1995). Additionally, factors such as injuries, aging, and other life stressors can exert additional psychological pressure on athletes. Unfortunately, many athletes tend to avoid seeking support for their mental well-being due to factors like shame, lack of awareness of the impact of psychological health on performance, and a perception that seeking help is a sign of weakness (Gulliver et al., 2012). Over time, this accumulated stress can manifest as impulsive behavior and, to some extent, risky behavior.

Secondly, this study pioneers the exploration of the relationship between mindfulness and athlete impulsive behavior. The results indicate a significant positive correlation between mindfulness and self-reflection/coping effectiveness (see Figure 2), supporting the findings of Mösler et al. (2022) and Kaiseler et al. (2017). Within the model developed in this study, mindfulness exerts the greatest influence on self-reflection, followed by coping effectiveness. Self-reflection and coping effectiveness mediate the relationship between mindfulness and impulsive behavior. As shown in Figure 2, these variables explain 35% of the variance in impulsive behavior. This study provides a valuable pathway for investigating the relationship between mindfulness and impulsive behavior, delving into the role of self-reflection at the psychological level. Additionally, it lends theoretical support to the study of mindfulness in athletes.

### 5.2 Practical implications

Considering the positive impact of mindfulness on self-reflection and coping effectiveness, as well as its indirect influence on reducing impulsive behavior in athletes, this study offers the following recommendations.

At the national level, the National Sports Bureau can take action on three fronts. Firstly, promote mindfulness training through extensive awareness campaigns. Launch large-scale publicity campaigns using various media platforms such as television, radio, social media, and ad slots during sporting events to emphasize the importance of mindfulness in sports (Brumitt et al., 2018). Collaborate with internationally renowned mindfulness training organizations and invite experts to conduct mindfulness courses in China, ensuring the introduction of the latest and most cutting-edge mindfulness education methods and materials. Recognize and reward teams or athletes who have successfully adopted mindfulness practices and achieved significant results, further motivating others to participate in mindfulness training. Secondly, develop and promote strategies for integration. Encourage sports teams at all levels to incorporate mindfulness training into their training routines, making it a standardized component of training. Establish dedicated teams to create, review, and disseminate official mindfulness materials and guidelines, ensuring their scientific rigor and practicality. Create an online platform offering mindfulness training videos, audio resources, and documents, making it convenient for coaches and athletes to learn and practice mindfulness anytime, anywhere. Thirdly, implement ongoing monitoring and evaluation. Set up standardized assessment systems, such as specially designed questionnaires and psychological tests, to regularly assess athletes’ impulsive behavior and the effectiveness of mindfulness training (van Rensburg et al., 2015). Establish a central database to collect training, competition, and mental health data from all athletes, enabling the analysis of the effects of mindfulness training on athletic performance. Based on the data collected and analysis results, make timely adjustments to the content and methods of mindfulness training to ensure its continued effectiveness and adaptability.

At the coach level, implement mindfulness training strategies in three ways. Firstly, actively engage in and practice mindfulness training. Participate in regular mindfulness workshops or seminars to stay updated on the latest research and applications of mindfulness in sports. Incorporate daily meditation and mindfulness exercises into your routine, building a solid foundation for guiding athletes in the future. Regularly exchange mindfulness practice experiences and insights with other coaches, exploring how to better integrate mindfulness into sports training. Secondly, integrate mindfulness into daily training. Before each training session, guide athletes through brief meditation or breathing exercises to help them focus their attention and prepare mentally. Utilize breaks in training, such as between skill and physical training, to allow athletes to engage in short mindfulness relaxation sessions to help them relax both physically and mentally. At the end of training, conduct a few minutes of relaxation meditation to help athletes alleviate fatigue and consolidate training content. Thirdly, provide regular mindfulness feedback to athletes. For example, schedule a weekly dedicated psychological communication session to listen to athletes’ concerns and provide feedback on impulsive behavior. Conduct individual case analyses for athletes displaying impulsive behavior, gaining deeper insights into the underlying psychological factors, and offering corresponding mindfulness adjustment recommendations. Additionally, organize monthly team-sharing sessions where athletes can share their gains and experiences in mindfulness practice, learn together, and motivate each other.

Lastly, at the athlete’s individual level, implement strategies in three ways. Firstly, actively engage in and deepen mindfulness practice. Establish a consistent daily meditation and mindfulness practice schedule, such as 10–15 min of meditation every morning or before bedtime. Utilize mindfulness-related mobile apps for guided meditation, making it convenient to practice mindfulness anytime, anywhere. Participate in collective meditation activities with teammates, encouraging and supporting each other’s progress (Amnie, 2018). Secondly, engage in self-monitoring and reflection. Maintain a mental diary, recording impulsive behaviors, emotional responses, and daily experiences with mindfulness practice. This can help athletes gain a clearer understanding of their emotional patterns and behavioral habits. Set aside time each week or month for self-review and assessment of behavior, emotions, and progress in mindfulness practice. Thirdly, seek support and help promptly. When feeling that emotions or impulsive behavior are becoming difficult to control, communicate with the coach for professional advice and guidance. When necessary, consider seeking help from mental health experts through psychological counseling to gain a deeper understanding of emotional and behavioral patterns and receive more specialized guidance and recommendations. Share experiences and challenges with teammates, listen to their opinions and suggestions, and collectively explore how to better utilize mindfulness to improve performance and reduce impulsive behavior.

In summary, these specific recommendations for the National Sports Bureau, coaches, and athletes themselves will greatly contribute to mitigating impulsive behavior in athletes.

## 6 Conclusion

According to the research objectives, the findings of this study reveal that mindfulness, self-reflection, and coping effectiveness play crucial roles in controlling impulsive behavior among athletes. Specifically, mindfulness positively contributes to reducing impulsive behavior among athletes, and this effect is mediated by two intermediary variables: self-reflection and coping effectiveness. Through mindfulness training, athletes can better navigate competitive pressure, enhance self-reflection skills, and further bolster coping effectiveness. This series of positive effects aids athletes in better understanding and managing their emotions and behaviors, ultimately reducing impulsive actions.

Therefore, we strongly recommend athletes consider incorporating mindfulness training into their daily routines and lives as a significant preventive measure against impulsive behavior. Furthermore, coaches and relevant sports organizations should prioritize the mental health of athletes. Providing mental health support, training in emotional management skills, and guidance in mindfulness training can help athletes better cope with challenges, reduce competitive pressure, and minimize impulsive behavior. In the end, these efforts not only benefit athletes’ personal growth and athletic performance but also contribute to shaping a healthier and more positive sports culture.

This study has several limitations that should be acknowledged. Firstly, the study did not employ random sampling, posing a slight limitation in the sample selection (Diotaiuti et al., 2020), which could introduce some bias into the sample. To ensure broader representativeness of the sample, future research should consider the use of random sampling techniques. Moreover, the specificity of the sample (Diotaiuti et al., 2021), limited to the central China region, should be noted. Secondly, this study did not incorporate moderator variables, such as the type of sport or the athletes’ anxiety levels, into the research model. Additionally, athletes selected were not queried beforehand about whether they had received mindfulness training (Diotaiuti et al., 2023). Therefore, future research should include these moderator variables to obtain a more comprehensive understanding of the subject. Additionally, considering that an increased sample size could enhance the study’s persuasiveness, it is recommended that future research expand the sample range.

## Data availability statement

The raw data supporting the conclusions of this article will be made available by the authors, without undue reservation.

## Ethics statement

The studies involving humans were approved by the Ethics Committee of the School of Physical Education of Hunan University of Science and Technology (No. ECBPEHNUST 2022/0012). The studies were conducted in accordance with the local legislation and institutional requirements. Written informed consent for participation in this study was provided by the participants’ legal guardians/next of kin.

## Author contributions

PL: Conceptualization, Investigation, Methodology, Writing – original draft, Writing – review & editing. HJ: Investigation, Resources, Writing – original draft, Writing – review & editing. HW: Conceptualization, Project administration, Writing – original draft, Writing – review & editing. JT: Investigation, Writing – original draft, Writing – review & editing.
